# The Memorial to Sir Andrew Clark, Bart

**Published:** 1895-09-21

**Authors:** 


### THE MEMORIAL TO SIR ANDREW CLARK, BART.

The best friends of the London Hospital cannot congratulate the House Committee on their proceedings at the special committee on Monday, summoned to consider our article on "A Mismanaged Memorial." The accommodation at the London Hospital for the isolation of erysipelas and other similar cases has been for years notoriously inadequate. When Sir Andrew Clark died the London Hospital authorities realised that the occasion of his death might be utilised to raise £12,000 to build a new wing, in which adequate accommodation for these cases could be provided. At the very first meeting they pledged the Memorial Committee to this scheme, and feelings of regard for the late Sir Andrew Clark and his great services to the London Hospital should have secured that these new buildings, to be known as the Andrew Clark Memorial Wing, should have been promptly erected, whatever sum might be raised by the Memorial Committee. As the matter stands at present the memorial wing has been reduced to insignificant proportions, and one-third of it is to be devoted to accommodation for the porters. The London Hospital has moreover greatly benefited financially by the publicity attending the proceedings of the Andrew Clark Memorial Committee, and there is evidence that the House Committee had abundance of funds for building purposes, seeing that they have sanctioned an entirely new project, and are at present erecting a new wing for the accommodation of the nurses, which is to cost £9,000 at least.

In our view the whole proceedings in connection with the Andrew Clark Memorial have been grievously mismanaged, and the explanation now published by the House Committee points to an absence of generosity on the part of the authorities of the London Hospital to the memory of the great physician who did so much to raise the reputation of the institution, which is unique in its short-sightedness and ingratitude. We therefore feel that justice demands that the £3,000 now available should not be sunk in buildings connected with the London Hospital, which will be largely devoted to accommodation for the porters. The Memorial Committee who represent the public, or at any rate those members of it who are not attached to the London Hospital, should insist in all the circumstances that the form of the memorial shall be changed. It was evidently a mistake to permit the London Hospital to attempt to make capital out of the sentiment of public gratitude and affection engendered by the life work of the late Sir Andrew Clark. The memorial ought from the outset to have taken a more personal form, distinct from any existing charity, and the original error must now be rectified. An exhibition in pathology attached to the medical school of the London Hospital, or to the new University for London, entitled the Andrew Clark Memorial, would best meet the case.

We have always upheld the authorities of the London Hospital when we have felt them to be in the right, but on the present occasion it would be an injustice not to protest against the action of the House Committee, who have shown so little sense of the proprieties of the case. The best interests of the patients have for years demanded the erection of such a block as that which they originally contemplated as a memorial to Sir Andrew Clark. Having the means at their disposal, they have preferred not to expend them upon this object, and their conduct is calculated to do a great injury to the popularity of the London Hospital. Nor is this all, for their proceedings have tended to create an impression that the memory of Sir Andrew Clark has received scant consideration at their hands, and much pain has in consequence been caused to the many friends of the late illustrious President of the Royal College of Physicians. In all the circumstances the subscribers to the memorial must combine to defeat the present proposal of the House Committee of the London Hospital, and to secure a memorial to the late Sir Andrew Clark which shall more worthily represent the widespread feelings of respect and affection felt for the man by his contemporaries and friends.

